# ID 293 - Inclusão da Modalidade Diretrizes na Epidemiologia e Serviços de Saúde: revista do SUS

**DOI:** 10.5327/2237-9622.2025.v34s1.293

**Published:** 2025-11-25

**Authors:** Marilia Mastrocolla de Almeida Cardoso, Jorge Otávio Maia Barreto, Dalila de Carvalho Silva Gonzaga, Aline Vieira de Lima, Taís Freire Galvão

## Abstract

**Introdução:**

A proposta da presente iniciativa é apresentar a nova modalidade de publicação na Epidemiologia e Serviços de Saúde: revista do SUS (RESS). A RESS abrange o campo da saúde coletiva que apresenta evidências relevantes ao Sistema Único de Saúde (SUS). Em 2024, a RESS ampliou seu escopo e incluiu a modalidade "Diretrizes", para publicar produtos que orientam condutas no âmbito do SUS. Tal ampliação contribui para a disseminação de avaliações de tecnologias em saúde, incluindo protocolos clínicos e diretrizes terapêuticas.

A RESS é um periódico científico de acesso aberto com publicação contínua e sem custos aos autores e leitores, mantido pela Secretaria de Vigilância em Saúde e Ambiente do Ministério da Saúde (SVSA/MS). A RESS é indexada nas principais bases, como MEDLINE, SciELO, Scopus, Embase, LILACS, Web of Science e PubMed Central.

Todos os artigos publicados na RESS ficam disponíveis na modalidade acesso aberto e são traduzidos gratuitamente para o inglês, o que favorece a democratização do conhecimento científico nacional e internacionalmente e fortalece a produção científica brasileira voltada para o aprimoramento dos serviços oferecidos pelo SUS. Os títulos e resumos dos artigos também são traduzidos para o espanhol, o que contribui para a disseminação das pesquisas na América Latina.

Por meio das redes sociais, os artigos publicados são disseminados ao público em geral, com a participação dos autores nessa disseminação. Os manuscritos são também divulgados no blogue SciELO em Perspectiva, por meio de texto de cunho jornalístico, com a colaboração dos autores, e preparação de vídeo com os principais pontos da pesquisa. Outra importante ação de divulgação e fomento às publicações é o Prêmio RESS Evidencia, que reconhece e homenageia o melhor artigo original publicado na revista a cada ano.

**Método:**

Pretende-se apresentar vídeo do conteúdo do site da RESS, com uma simulação rápida de submissão e processamento, e as postagens das redes sociais para que os participantes possam conhecer as formas de divulgação oferecidas pela revista.

Conexão da internet, computador, projetor de imagem em tela grande e equipamento de som para apresentação dos vídeos.

